# An Optimized Damping Grey Population Prediction Model and Its Application on China’s Population Structure Analysis

**DOI:** 10.3390/ijerph192013478

**Published:** 2022-10-18

**Authors:** Xiaojun Guo, Rui Zhang, Houxue Shen, Yingjie Yang

**Affiliations:** 1 School of Science, Nantong University, Nantong 226019, China; 2 Institute of Artificial Intelligence, De Montfort University, Leicester LE1 9BH, UK

**Keywords:** population prediction, grey system, damping accumulated operator, grey Bernoulli model, whale optimization algorithm

## Abstract

Population, resources and environment constitute an interacting and interdependent whole. Only by scientifically forecasting and accurately grasping future population trends can we use limited resources to promote the sustainable development of society. Because the population system is affected by many complex factors and the structural relations among these factors are complex, it can be regarded as a typical dynamic grey system. This paper introduces the damping accumulated operator to construct the grey population prediction model based on the nonlinear grey Bernoulli model in order to describe the evolution law of the population system more accurately. The new operator can give full play to the principle of new information first and further enhance the ability of the model to capture the dynamic changes of the original data. A whale optimization algorithm was used to optimize the model parameters and build a smooth prediction curve. Through three practical cases related to the size and structure of the Chinese population, the comparison with other grey prediction models shows that the fitting and prediction accuracy of the damping accumulated–nonlinear grey Bernoulli model is higher than that of the traditional grey prediction model. At the same time, the damping accumulated operator can weaken the randomness of the original data sequence, reduce the influence of external interference factors, and enhance the robustness of the model. This paper proves that the new method is simple and effective for population prediction, which can not only grasp the future population change trend more accurately but also further expand the application range of the grey prediction model.

## 1. Introduction

Population and environment are coupled to form a mutually restrictive and interdependent organic whole. The population has always been a key issue in the development of human society, which directly affects environmental change, economic development and social stability [1]. Only by properly handling the relationship between population, resources and environment can we promote the sustainable development of society and realize the comprehensive and coordinated development of man and nature [2]. At the same time, in the relationship between economic development, the population, as an index to measure the degree of social progress, can reflect the economic development level of a region. From an international perspective, the trend of world population development is an important basis for the United Nations to formulate a global population strategy. Similar to other countries, the formulation of basic state policies, the arrangement of labor and employment, the development of social welfare, and even the standards of national economic and social development strategies in China are profoundly affected by population change. Especially under the situations that China is facing, such as the problems of an unbalanced birth rate, insufficient labor supply, weakening demographic dividend and serious aging trend, it is of great significance to understand the future population development trend of China, accurately grasp the population status, and scientifically predict the future population number for the scientific decision-making of population policy and the construction of a harmonious society.

A population forecast is a reliable and accurate forecast of the future population development trend based on the existing information, so as to make population activities and economic development move forward in a favorable direction. Choosing a reasonable model to accurately describe and forecast population and various population indicators has a decisive significance for formulating a suitable plan for population development which, coordinated with social and economic development, can provide scientific decision-making basis for economic and social development. Graunt and Erle et al. studied population prediction 300 years ago, focusing on the analysis and research from the perspective of age distribution and population evolution [3]. Later, Leslie et al. began to pay attention to the influence of deterministic models on population prediction and introduced population dynamics into population prediction [4]. With the rapid growth of the world population, the impact of population on society is increasing day by day. The population problem has become one of the major issues causing concern for the world. Common models for population prediction include the differential equation model, Leslie model, stochastic prediction model, mathematical statistics method, grey model, BP neural network model, linear time series model, deep learning models, etc. [5,6,7,8]. For the convenience of a comparative study, we summarize the mechanisms, assumptions, merits and drawbacks of mainstream population prediction methods, as shown in Table 1.

Each population prediction method has its own advantages and disadvantages, therefore it needs to make a choice according to the specific analysis of the problem. For example, although the prediction accuracy of the differential equation model is high, with the increase in variables, the equation will become more complicated and the difficulty of solving it will increase accordingly. The Leslie matrix model is more suitable for large time ranges and large time-span prediction problems. However, due to the different birth and death rates of different age groups, it is not suitable to analyze population growth under natural conditions, which limits the actual use of the Leslie matrix model. Although population development is a dynamic development process, the linear programming model is only suitable for the prediction of problems with a linear relationship, and the population problem inevitably has a nonlinear relationship, therefore the linear model has a limited role in population prediction. Although the grey prediction model reflects the regularity of data, it has the limitation that it cannot completely reflect the influence of various irregular social factors on the prediction index. The improvement of the grey prediction model, which not only gives full play to the regularity of data but also avoids the influence of irregular social factors on indicators, has become a research hotspot.

After professor Deng Julong first proposed the grey system theory in 1982, the grey system theory was gradually enriched and improved after 40 years of development [9]. As a new system analysis method to solve the problem of “few data, poor information and uncertain problem “, it has gradually become one of the main schemes to study uncertain systems. The inherent laws of the data sequence are fully mined to make scientific and quantitative predictions, so as to reveal the future development trend of the system [10]. Since the grey prediction model is not strict about the sample size and the probability distribution of the original sequence, it has a unique advantage in the prediction of small sample data and effectively solves a large number of practical problems in production and life. At present, a large number of research results with practical value have been obtained in various dynamic systems such as public health [11,12], transportation [13,14,15], energy development [16,17,18], air quality [19,20] and so on. At the same time, many scholars have carried out abundant optimization studies on the traditional grey prediction model based on the characteristics of the grey prediction model [21], the construction of the initial value and background value of the model [22,23], the evolution of the accumulative generation operator [24,25], the expansion of the modeling equation [26], and the combined prediction with other models [27].

In the past, many scholars have undertaken a lot of exploration into the grey system theory. In particular, in the field of population prediction, most of the research is aimed at the traditional GM(1,1) model, which is formally expressed as a system with linear characteristics. However, in practical applications, most systems have mostly nonlinear characteristics and the dynamic changes of population time series data are the result of the synergistic effects of many factors, such as primary, secondary, direct, indirect, known, unknown, obvious and implicit, which are interrelated and mutually restrictive. These factors, which are partly known and partly unknown, interact with each other to determine the grey amount of reality—the total population. As a dynamic development process, population evolution inevitably has nonlinear characteristics, therefore only relying on a linear prediction model is not suitable for all systems. The Nonlinear Grey Bernoulli Model (referred to as NGBM), as a generalized nonlinear grey model, is an inevitable trend of research and development. Therefore, this paper considers using the NGBM model to model the population system, which is characterized by multi-factor control, complex evolution law, and uncertain data fluctuation, etc. Through a nonlinear model, the evolution law of the population system can be more effectively fitted.

As a general grey prediction model, the structure of the NGBM model changes with the power exponent. When the power exponent is zero, it is the traditional GM(1,1) model. When the power exponent is two, the model is a special grey Verhulst model [28]. Meanwhile, the power exponent of the NGBM model has the flexibility to change appropriately with the trend of the original sequence. When the value of the power exponent is appropriate, the NGBM model can achieve high prediction accuracy. Therefore, the NGBM model should have more ideal results than the traditional linear model to predict the future population. However, in practice, it is still found that the fitting and prediction effect of this model are unstable. In order to make the model capture the changing trend of different original behavior sequences more closely, make full use of the value of new information, and further improve the stability and computational efficiency of the model, a new accumulation operator can be applied in the NGBM model. A large number of studies were conducted on how to properly preprocess the original data and many cumulative operators suitable for different characteristics of the original data series were proposed, such as fractional order accumulation [29], complex order accumulation [30], seasonal accumulation [31,32], new information priority accumulation [33], etc.

It is mentioned in the grey system theory that if the original data have an exponential growth trend, they are more in line with the requirements of grey system modeling. The population can be regarded as a kind of data with exponential properties and saturated growth trends, therefore it is very suitable to be processed by the NGBM model. However, in the process of predicting this kind of data, the traditional accumulation operator will lead the prediction result to increase too much in the short term and there will be a large deviation between the prediction result and the reality. At the same time, owing to the traditional accumulation operator, the model cannot accurately control the mutation points in the data series, resulting in it not being able to accurately describe the disadvantages of the population change trend. By using new accumulation methods, such as fractional order or complex order accumulation, the order of data accumulation can be extended from the integer range to the real or complex number field, which can greatly increase the prediction accuracy of the model and enhance the goodness of fit with the real data. However, these methods are too complex in their calculations, which is not conducive to application and theoretical analysis. Therefore, in order to avoid the phenomenon of exponential growth or rapid decline of data change in the process of prediction and make the prediction result conform to the law of population growth as much as possible, Liu Lianyi et al. proposed a kind of damping accumulated operator with a damping trend, which makes it easy to be concise and easy to understand and popularize [34]. By giving different weights to the information at different time points, the proportion of new information in the system is higher than that of old information, so as to highlight the role of new information in the sequence and effectively enhance the smoothness of the prediction data sequence and the stability of the prediction model. Through research, it was found that the combination of the damping accumulated operator and the traditional GM(1,1) model provides better performance compared with the traditional grey prediction model.

In this paper, the damping accumulated generation operator is combined with a nonlinear grey Bernoulli model to form the damping accumulated nonlinear grey Bernoulli model (referred to as DA–NGBM). On the basis of further improving the prediction accuracy and universality of the nonlinear model, the stability of model prediction and the reliability of prediction results are enhanced effectively. After fully studying the model, three cases related to the population size and structure of China further proved that the damping accumulated operator can effectively optimize the prediction efficiency of the original NGBM model and it can not only grasp the changes of the original sequence of different trends more accurately but is also suitable for the study of population direction prediction. Thus, it will really expand the application scope of the grey prediction model.

The rest of this article is organized as follows. In the second section, the traditional NGBM model and its solving process are reviewed, as well as the damped accumulated generating operator and related properties. In the third part, a new DA–NGBM model is constructed and its parameter estimation method, error reduction and perturbation boundary analysis are discussed. In the fourth part, three cases about the population size and structure of China are selected to verify the validity of the new model by comparing it with other traditional models. The fifth part makes a visual analysis of the testing results of the four models and discusses the performance of six kinds of prediction models for three actual cases, and then analyzes the prediction advantages of the DA–NGBM model in detail. Finally, the sixth part gives the conclusion of this paper and the prospects for future work.

## 2. The Definition of the Damping Accumulated Generating Operator

### 2.1. Traditional Grey Accumulation Generating Operator and Its Generating Sequence

The real data, to a great extent, reflect the actual situation of a certain problem in the objective world. Even if the type of data is in a discrete form or there is no law that can be found intuitively, as a direct manifestation of the behavior of the system, it must contain the deep evolution law of the system. In order to mine valuable and usable information from discrete and chaotic original data, the concepts of accumulation operator and accumulation generating sequence are proposed. By applying the summation operator on the original sequence items of the system, the intuitive data with irregular and large fluctuation can be transformed into usable data with obvious regularity and a linearly increasing relationship. Such operations facilitate the subsequent model building and the data have a powerful ability to display the actual behavior of the system, so as to more clearly highlight the evolution process and internal laws of the system.

**Definition** **1**  **([35]).** *Suppose*$X^{\left(0\right)}=(x^{\left(0\right)}(1),x^{\left(0\right)}(2),\cdot \cdot \cdot ,x^{\left(0\right)}(n))$*is the non-negative original data sequence,* $X^{\left(0\right)}D=(x^{\left(0\right)}(1)d,x^{\left(0\right)}(2)d,\cdot \cdot \cdot ,x^{\left(0\right)}(n)d)$ *is the first-order accumulation sequence of*  $X^{\left(0\right)}$*,* D *is the sequence operator, where*

(1) $$x^{\left(0\right)}(k)d=\sum_{i=1}^{k}x^{\left(0\right)}(i),\begin{array}{cc} & k=1,2,\cdot \cdot \cdot ,n \end{array}$$

D *is the first-order cumulative generating operator of* $X^{\left(0\right)}$*, and is denoted as 1-AGO.*

(2) $$x^{\left(r\right)}(k)=\sum_{i=1}^{k}x^{\left(r-1\right)}(i),\begin{array}{cc} & k=1,2,\cdot \cdot \cdot ,n \end{array}$$

*is called the r-order accumulation generated sequence of the original sequence* $X^{\left(0\right)}$.

The traditional grey accumulation generating operator can effectively improve the smoothness of the sequence, reduce the fluctuation among the discrete data, enhance the regularity of the data, and ensure the accuracy and stability of the grey prediction model fitting results and prediction results. At the same time, as the operator of equal weight addition, it fails to distinguish between new and old data sequences at each time point, and the data at each time point has the same proportion in the system, which will lead to a decrease in the fitting accuracy of the prediction model. In addition, assigning the old and new information with the same weight to the fitting sequence cannot highlight the “new information priority principle” required by the grey system theory. Moreover, the new information with closer time distance and more accurate and timely practical significance cannot be effectively played, which limits the prediction effect of the model to a certain extent.

**Definition** **2**  **([35]).** *Suppose*$X^{\left(0\right)}$*is the original data sequence,* $X^{\left(1\right)}$ *is the first-order accumulative generated sequence of* $X^{\left(0\right)}$*,* $Z^{\left(1\right)}=\left(z^{\left(1\right)}(1),z^{\left(1\right)}(2),\cdot \cdot \cdot ,z^{\left(1\right)}(n)\right)$ *is the action sequence of the adjacent mean value operator of* $X^{\left(1\right)}$*,*

(3) $$x^{\left(0\right)}(k)+az^{\left(1\right)}(k)=b{\left[z^{\left(1\right)}(k)\right]}^{\alpha }$$

*is called the nonlinear grey Bernoulli model, where* a *is the development coefficient,* b *is the grey action quantity,* α *is the power exponent, and*

$$z^{\left(1\right)}(k)=\frac{1}{2}(x^{\left(1\right)}(k-1)+x^{\left(1\right)}(k)),\begin{array}{cc} & k=2,3,\cdot \cdot \cdot ,n \end{array}.$$

The solution process of the NGBM model is described as follows:

**Step 1.** Suppose $X^{\left(0\right)}=(x^{\left(0\right)}(1),x^{\left(0\right)}(2),\cdots ,x^{\left(0\right)}(n))$ is the non-negative original data sequence, $X^{\left(1\right)}=(x^{\left(1\right)}(1),x^{\left(1\right)}(2),\cdots ,x^{\left(1\right)}(n))$ is the first-order accumulative generated sequence of $X^{\left(0\right)}$ and $Z^{\left(1\right)}=(z^{\left(1\right)}(2),z^{\left(1\right)}(3),\cdots ,z^{\left(1\right)}(n))$ is the action sequence as described in definition 2 above.

**Step 2.** Suppose $X^{\left(0\right)}$, $X^{\left(1\right)}$ and $Z^{\left(1\right)}$ are as described in Step 1, the grey differential equation of the NGBM model is

(4) $$x^{\left(0\right)}(k)+az^{\left(1\right)}(k)=b{\left(z^{\left(1\right)}(k)\right)}^{\alpha },$$

and the whitening differential equation can be expressed as

(5) $$\frac{dx^{\left(1\right)}(t)}{dt}+ax^{\left(1\right)}(t)=b{\left(x^{\left(1\right)}(t)\right)}^{\alpha }$$

By solving the above differential equations, the time response of the NGBM model can be obtained as

(6) $$x^{\left(1\right)}(t+1)={\left\{e^{(\alpha -1)at}\cdot \left[(1-\alpha )\int b\cdot e^{(1-\alpha )at}dt+c\right]\right\}}^{\frac{1}{1-\alpha }}={\left(\frac{b}{a}+c\cdot e^{-(1-\alpha )at}\right)}^{\frac{1}{1-\alpha }}$$

**Step 3.** The parameter sequence $\left[\begin{array}{c} \hat{a}\\ \hat{b} \end{array}\right]$ of the model can be estimated by least squares based on the principle of minimum error sum of squares

(7) $$\left[\begin{array}{c} \hat{a}\\ \hat{b} \end{array}\right]={\left(B^{T}B\right)}^{-1}B^{T}Y.$$

where $B=\left[\begin{array}{cc} -z^{\left(1\right)}(2) & {\left(z^{\left(1\right)}(2)\right)}^{\alpha }\\ -z^{\left(1\right)}(3) & {\left(z^{\left(1\right)}(3)\right)}^{\alpha }\\ \vdots & \vdots \\ -z^{\left(1\right)}(n) & {\left(z^{\left(1\right)}(n)\right)}^{\alpha } \end{array}\right]$, $Y=\left[\begin{array}{c} x^{\left(0\right)}(2)\\ x^{\left(0\right)}(3)\\ \vdots \\ x^{\left(0\right)}(n) \end{array}\right]$.

**Step 4.** By substituting the parameters $\hat{a}$ and $\hat{b}$ into the time response function (6) and setting ${\hat{x}}^{\left(1\right)}(1)=x^{\left(0\right)}(1)$, the time response of the original sequence can be obtained as

(8) $${\hat{x}}^{\left(1\right)}(k+1)={\left\{\frac{\hat{b}}{\hat{a}}+\left[{\left(x^{\left(0\right)}(1)\right)}^{1-\alpha }-\frac{\hat{b}}{\hat{a}}\right]e^{-\left(1-\alpha \right)\hat{a}k}\right\}}^{\frac{1}{1-\alpha }}$$

where k=1,2,⋯,n,⋯, ${\hat{x}}^{\left(1\right)}(k+1)$ is the fitting value of the k+1 moment, from which the sequence ${\hat{X}}^{\left(1\right)}$ can be obtained.

**Step 5.** The sequence ${\hat{X}}^{\left(1\right)}$ is reduced by the first order, from which the fitting sequence of the original data can be obtained as ${\hat{X}}^{\left(0\right)}=\{{\hat{x}}^{\left(0\right)}(1),{\hat{x}}^{\left(0\right)}(2),\cdots ,{\hat{x}}^{\left(0\right)}(n)\}$, and the predicted value of the model is ${\hat{x}}^{\left(0\right)}(n+1)$, ${\hat{x}}^{\left(0\right)}(n+2)$,⋯, where

(9) $${\hat{x}}^{\left(0\right)}(k+1)={\hat{x}}^{\left(1\right)}(k+1)-{\hat{x}}^{\left(1\right)}(k),\begin{array}{cc} & k=1,2,\cdot \cdot \cdot \end{array}$$

### 2.2. Damping Accumulated Generating Operator and Its Generating Sequence

The traditional grey cumulative generating operator (abbreviated as AGO) can solve the problem that the data in the original behavior sequence are discrete and have no intuitive rules. However, since the cumulative operator gives the same weight to the behavioral data at each time point in the system, AGO cannot distinguish the difference between the old and new information. This will lead to a large deviation between the prediction model and the real data in the process of fitting. In order to solve these problems and further improve the prediction accuracy of the grey prediction model, the damping accumulated operator is specially introduced. By setting damping parameters, the weights of old and new information in the system can be distinguished, which makes the model perfectly conform to the “new information priority principle”, meaning more satisfactory prediction results can be obtained.

**Definition** **3**  **([34]).** *Suppose*$X^{\left(0\right)}=(x^{\left(0\right)}(1),x^{\left(0\right)}(2),\cdot \cdot \cdot ,x^{\left(0\right)}(n))$*is the non-negative original data sequence, its* ζ *-order damping accumulation sequence is defined as* $X^{\left(\zeta \right)}=(x^{\left(\zeta \right)}(1),x^{\left(\zeta \right)}(2),\cdot \cdot \cdot ,x^{\left(\zeta \right)}(n))$*, where*

(10) $$x^{\left(\zeta \right)}(k)=\sum_{i=1}^{k}\frac{x^{\left(0\right)}(i)}{\zeta^{i-1}},\begin{array}{cc} & k=1,2,\cdot \cdot \cdot ,n \end{array}$$

 *is called the* ζ *-order damping summation operator of the sequence* $X^{\left(0\right)}$*, the damping coefficient* $\zeta \in \left(0,1\right]$.

Due to the $x^{\left(\zeta \right)}(n)=\sum_{i=1}^{n}\frac{x^{\left(0\right)}(i)}{\zeta^{i-1}}=x^{\left(0\right)}(1)+\frac{x^{\left(0\right)}(2)}{\zeta }+\cdot \cdot \cdot +\frac{x^{\left(0\right)}(n)}{\zeta^{n-1}}$ matrix form of the sequence generated by ζ-order damping accumulation can be expressed as

(11) $$\left(x^{\left(\zeta \right)}(1),x^{\left(\zeta \right)}(2),\cdots ,x^{\left(\zeta \right)}(n)\right)=\left(x^{\left(0\right)}(1),x^{\left(0\right)}(2),\cdots ,x^{\left(0\right)}(n)\right)\left(\begin{array}{cccc} 1 & 1 & \cdots & 1\\ 0 & \frac{1}{\zeta } & \cdots & \frac{1}{\zeta }\\ \vdots & \vdots & \cdots & \vdots \\ 0 & 0 & \cdots & \frac{1}{\zeta^{n-1}} \end{array}\right)$$

When ζ=1, it can be found that the ζ-order damping accumulated operator is equivalent to 1-AGO and the ζ-order damping accumulation sequence of the original sequence is equivalent to the traditional first-order accumulation generation sequence proposed in definition 1.

The damping accumulated operator can be used to optimize the traditional accumulation operator. Although 1-AGO makes the original discrete data with larger floating have a linear growth relationship easier to observe through data addition, the equal-weight addition of the data at each time in the original sequence ignores the difference of information between the old and new data and fails to make full use of the distance to study the practical value of the new data at a relatively recent time. Therefore, the damping accumulated operator is optimized in this aspect. By introducing the concept of damping parameters, the data information at each time point has its own weight so as to carry out differential treatment of the influence of the old and new information in the system. Therefore, the newer information in the system accounts for a larger proportion of influence, thus affecting the model fitting efficiency, and improving the prediction ability of the model.

**Property** **1**  **([34]).** *Let*$x^{\left(\zeta \right)}(k)$ (k=1,2,⋅⋅⋅,n) *be the* ζ *-order damping accumulated operator, then the damping accumulation sequence* $X^{\left(\zeta \right)}=(x^{\left(\zeta \right)}(1),x^{\left(\zeta \right)}(2),\cdot \cdot \cdot ,x^{\left(\zeta \right)}(n))$ *monotonically increases with respect to the time variable* k.

**Proof** **of** **Property** **1.** For $\forall k\in \left[1,n\right]$, we have

$$x^{\left(\zeta \right)}(k+1)-x^{\left(\zeta \right)}(k)=\sum_{i=1}^{k+1}\frac{x^{\left(0\right)}(i)}{\zeta^{i-1}}-\sum_{i=1}^{k}\frac{x^{\left(0\right)}(i)}{\zeta^{i-1}}=\frac{x^{\left(0\right)}(k+1)}{\zeta^{k}}$$

Since $X^{\left(0\right)}$ is a non-negative sequence, $\zeta \in \left(0,1\right]$, so $x^{\left(\zeta \right)}(k+1)-x^{\left(\zeta \right)}(k)>0$ can be obtained. The proof is that the time variable is monotonically increasing. That is, $X^{\left(\zeta \right)}$ is monotonically increasing with respect to the time variable k. □

In the model, the more recent the research year, the more information contained in the new data, and the more research value. However, population systems generally have an evolutionary trend that cannot be mutated, and with the increase in time, the annual population generally shows a law of slow growth or decline. The connotation of this nature is basically consistent with the actual situation of the population system.

**Property** **2**  **([34]).** *The value of*$x^{\left(\zeta \right)}(k)$*in the damping accumulation sequence* $X^{\left(\zeta \right)}$ *is negatively correlated with the magnitude of the parameter* ζ.

**Proof** **of** **Property** **2.** For $\forall \zeta_{1},\zeta_{2}\in \left(0,1\right]$, set damping parameters $\zeta_{1}<\zeta_{2}$, then $\frac{1}{\zeta_{1}}>\frac{1}{\zeta_{2}}$, and $\frac{1}{\zeta_{1}{}^{i}}>\frac{1}{\zeta_{2}{}^{i}},\;i=1,2,\cdot \cdot \cdot ,n$.

Due to the $x^{\left(\zeta_{1}\right)}(k)-x^{\left(\zeta_{2}\right)}(k)=\sum_{i=1}^{k}x^{\left(0\right)}(i)(\frac{1}{{\zeta_{1}}^{i-1}}-\frac{1}{{\zeta_{2}}^{i-1}})$, we can get to know $x^{\left(\zeta_{1}\right)}(k)>x^{\left(\zeta_{2}\right)}(k),\;k=1,2,\cdot \cdot \cdot ,n$.

That is, the value $x^{\left(\zeta \right)}(k)$ is negatively correlated with the magnitude of the parameter ζ. □

By setting the value of damping parameters, the proportion of each item in the original sequence in the damping accumulation sequence can be controlled. It can be found that the equal weights of the original sequence items are no longer added in the damping accumulation term, and the smaller the value ζ is, the greater the weight of the items in the original sequence in the new sequence will be, that is, it will occupy more proportion in the system research.

**Definition** **4.** *Suppose* $X^{\left(r\right)}=\{x^{\left(r\right)}(1),x^{\left(r\right)}(2),\cdots ,x^{\left(r\right)}(n)\}$ *is a non-negative time series, define*

(12) $$\Delta (i)=\left|x^{\left(r\right)}(i+1)-x^{\left(r\right)}(i)\right|$$

*is the information difference between* $x^{\left(r\right)}(i+1)$ *and* $x^{\left(r\right)}(i)$.

**Property** **3.** *Suppose* $X^{\left(\zeta \right)}$ *is the damping accumulated with the original data to generate the sequence and the information difference* Δ(k) *of the sequence* $X^{\left(\zeta \right)}$ *is the increasing function of the time variable* k *and the decreasing function of the damping parameter* ζ.

**Proof** **of** **Property** **3.** According to the definition of information difference and damping accumulated generating operator,

$$\Delta (k)=x^{\left(\zeta \right)}(k+1)-x^{\left(\zeta \right)}(k)=\left(x^{\left(0\right)}(1),x^{\left(0\right)}(2),\cdot \cdot \cdot ,x^{\left(0\right)}(k),x^{\left(0\right)}(k+1)\right)\left(\begin{array}{c} \begin{array}{c} 1-1\\ \frac{1}{\zeta }-\frac{1}{\zeta } \end{array}\\ \vdots \\ \frac{1}{\zeta^{k-1}}-\frac{1}{\zeta^{k-1}}\\ \frac{1}{\zeta^{k}}-0 \end{array}\right)=\frac{x^{\left(0\right)}(k+1)}{\zeta^{k}}.$$

Then, the information difference Δ(k) of $X^{\left(\zeta \right)}$ can be expressed in matrix form as

$$\left(\Delta (1),\Delta (2),\cdots ,\Delta (k)\right)=\left(x^{\left(0\right)}(2),x^{\left(0\right)}(3),\cdot \cdot \cdot ,x^{\left(0\right)}(k+1)\right)\left(\begin{array}{cccc} \frac{1}{\zeta } & 0 & \cdots & 0\\ 0 & \frac{1}{\zeta^{2}} & \cdots & 0\\ \vdots & \vdots & \cdots & \vdots \\ 0 & 0 & \cdots & \frac{1}{\zeta^{k}} \end{array}\right).$$

Obviously, when $\zeta \in \left(0,1\right]$, the information difference Δ(k) of the accumulated damping sequence $X^{\left(\zeta \right)}$ is the increasing function of the time variable k and the decreasing function of the damping parameter ζ. □

The larger the value of the time variable k is, the more recent the year represented by the data value is, which is consistent with the law of reality. This property can be expressed as the information difference between the newer data, which contains a more practical value and has more important research significance.

## 3. Damping Accumulated–Nonlinear Grey Bernoulli Model

### 3.1. Model Building

**Definition** **5.** *Suppose* X(0) *is original data sequence,* X(ζ) *is the sequence generated by the damping accumulated of* X(0)*, then*

(13) $$x^{\left(\zeta \right)}(k)-x^{\left(\zeta \right)}(k-1)+az^{\left(\zeta \right)}(k)=b{\left[z^{\left(\zeta \right)}(k)\right]}^{\alpha },\begin{array}{cc} & k=2,\cdots ,n \end{array}$$

*is called the damping accumulated–nonlinear grey Bernoulli model, where* $z^{\left(\zeta \right)}(k)=\frac{1}{2}(x^{\left(\zeta \right)}(k)+x^{\left(\zeta \right)}(k-1))$.

The modeling process of the DA–NGBM model is described as follows:

**Step 1.** Suppose $X^{\left(0\right)}=(x^{\left(0\right)}(1),x^{\left(0\right)}(2),\cdots ,x^{\left(0\right)}(n))$ is the non-negative sequence of the original data and the corresponding damped accumulative generating sequence $X^{\left(\zeta \right)}=(x^{\left(\zeta \right)}(1),x^{\left(\zeta \right)}(2),\cdots ,x^{\left(\zeta \right)}(n))$ of the original data and the adjacent to the mean operator action sequence $Z^{\left(\zeta \right)}=(z^{\left(\zeta \right)}(2),z^{\left(\zeta \right)}(3),\cdots ,z^{\left(\zeta \right)}(n))$ are defined in Section 2.

**Step 2.** Suppose $X^{\left(0\right)}$, $X^{\left(\zeta \right)}$ and $Z^{\left(\zeta \right)}$ are as described in Step 1, the grey differential equation of the DA–NGBM model is

(14) $$x^{\left(\zeta \right)}(k)-x^{\left(\zeta \right)}(k-1)+az^{\left(\zeta \right)}(k)=b{\left[z^{\left(\zeta \right)}(k)\right]}^{\alpha }$$

where a is the development coefficient and b is the grey action quantity. The whitening differential equation can be expressed as

(15) $$\frac{dx^{\left(\zeta \right)}(t)}{dt}+ax^{\left(\zeta \right)}(t)=b{\left(x^{\left(\zeta \right)}(t)\right)}^{\alpha }$$

After solving the above differential equation, the time response of the DA–NGBM model can be obtained as

(16) $$x^{\left(\zeta \right)}(t+1)={\left(\frac{b}{a}+c\cdot e^{(\alpha -1)at}\right)}^{\frac{1}{1-\alpha }}$$

**Step 3.** The parameter sequence $\left[\begin{array}{c} \hat{a}\\ \hat{b} \end{array}\right]$ of the model can be estimated by least squares, $\left[\begin{array}{c} \hat{a}\\ \hat{b} \end{array}\right]=\underset{a,b}{\arg\min}\left\{\sum_{k=1}^{n-1}{\left[\left(x^{\left(\zeta \right)}(k+1)-x^{\left(\zeta \right)}(k)\right)-\left(b{\left[z^{\left(\zeta \right)}(k)\right]}^{2}-az^{\left(\zeta \right)}(k)\right)\right]}^{2}\right\}$ based on the principle of minimum sum of error squares, we can obtain

(17) $$\left[\begin{array}{c} \hat{a}\\ \hat{b} \end{array}\right]={\left(B^{T}B\right)}^{-1}B^{T}Y$$

where $B=\left[\begin{array}{cc} -z^{\left(\zeta \right)}(2) & {\left(z^{\left(\zeta \right)}(2)\right)}^{\alpha }\\ -z^{\left(\zeta \right)}(3) & {\left(z^{\left(\zeta \right)}(3)\right)}^{\alpha }\\ \vdots & \vdots \\ -z^{\left(\zeta \right)}(n) & {\left(z^{\left(\zeta \right)}(n)\right)}^{\alpha } \end{array}\right]$, $Y=\left[\begin{array}{c} x^{\left(\zeta \right)}(2)-x^{\left(\zeta \right)}(1)\\ x^{\left(\zeta \right)}(3)-x^{\left(\zeta \right)}(2)\\ \vdots \\ x^{\left(\zeta \right)}(n)-x^{\left(\zeta \right)}(n-1) \end{array}\right]$.

**Step 4.** Substituting parameters $\hat{a}$ and $\hat{b}$ into the time response Function (16) and letting ${\hat{x}}^{\left(\zeta \right)}(1)=x^{\left(0\right)}(1)$, the time response formula of the original sequence can be obtained as

(18) $${\hat{x}}^{\left(\zeta \right)}(k+1)={\left\{\frac{\hat{b}}{\hat{a}}+\left[{\left(x^{\left(0\right)}(1)\right)}^{1-\alpha }-\frac{\hat{b}}{\hat{a}}\right]e^{-\left(1-\alpha \right)\hat{a}k}\right\}}^{\frac{1}{1-\alpha }}$$

where k=1,2,⋯,n,⋯, ${\hat{x}}^{\left(\zeta \right)}(k+1)$ is the fitting value of the k+1 moment, thus obtaining the sequence ${\hat{X}}^{\left(\zeta \right)}$.

**Step 5.** After the first-order reduction of sequence ${\hat{X}}^{\left(1\right)}$, the fitting sequence of the original data can be obtained as ${\hat{X}}^{\left(0\right)}=\{{\hat{x}}^{\left(0\right)}(1),{\hat{x}}^{\left(0\right)}(2),\cdots ,{\hat{x}}^{\left(0\right)}(n)\}$ and the predicted value of the model is ${\hat{x}}^{\left(0\right)}(n+1)$, ${\hat{x}}^{\left(0\right)}(n+2)$, ⋯. The reductive formula of the model is

(19) $${\hat{x}}^{\left(0\right)}(k)=\left\{\begin{array}{lll} x^{\left(0\right)}(1) & , & k=1\\ \left[{\hat{x}}^{\left(\zeta \right)}(k)-{\hat{x}}^{\left(\zeta \right)}(k-1)\right]\zeta^{k-1} & , & k=2,3\cdot \cdot \cdot \end{array}\right.$$

### 3.2. Parameter Estimation

Since the selection range of damping parameters is any real number within (0, 1), it will consume a lot of resources and time to manually calculate the prediction accuracy of the model corresponding to each parameter value to determine the value of damping ζ. For the indeterminate power exponent α in the DA–NGBM model, the optimal value still needs to be determined through a large number of calculations. Therefore, the estimation of an appropriate value can be optimized by using modern optimization algorithms. Considering the advantages of the heuristic algorithm, the whale optimization algorithm (WOA) currently has widely recognized computing power, therefore let the original sample data be divided into a training set and testing set, and the fitness function of the algorithm be set as the fitting prediction error of the DA–NGBM model. By finding the parameter corresponding to the global minimum of the objective function using the WOA algorithm, the damping parameter selection can be completed when the prediction model has the highest fitting accuracy.

A nonlinear optimization problem was established and the mean percentage error (MAPE) of the prediction model was used as the objective function

(20) $$\underset{\zeta }{\min}\;MAPE=\frac{1}{n}\sum_{i=1}^{n}\left|\frac{{\hat{x}}^{\left(0\right)}(i)-x^{\left(0\right)}(i)}{x^{\left(0\right)}(i)}\right|\times 100\%$$

The whale optimization algorithm is mainly inspired by the predation behavior of the humpback whale population and realizes the optimization search by simulating the scenes of encirclement, hunting and attacking the prey of the whale population. It assumes that the whale population size is N, the search space is *d*-dimensional, and the position of the *i*-th whale in *d*-dimensional space can be expressed as $x_{i}=\left(x_{i1},x_{i2},\cdots ,x_{id}\right)$, i=1,2,⋅⋅⋅,N.

The location of the prey corresponds to the global optimal solution of the problem and there is no prior condition for the global optimal position in the search space before solving the optimization problem. Therefore, it is assumed that the position of the optimal individual in the current population is the prey position and other individuals are surrounded by the optimal individual. Based on the random walk mechanism of whale group hunting, whale individuals update the position of the next generation according to the location information of each other in the group. According to the search results of the t time, they update the predation behavior of the t+1 time by using Equations (21) and (22).

(21) $$\vec{D}=|\vec{C}\cdot \vec{X^{*}}(t)-\vec{X}(t)|$$

(22) $$\vec{X}(t+1)=\vec{X^{*}}(t)-\vec{A}\cdot \vec{D}$$

where $\vec{D}$ is any position of whales in the hunting group, $\vec{X^{*}}(t)$ is the prey position and $\vec{A}$ and $\vec{C}$ are the coefficient vector, which is defined as follows:

(23) $$\vec{A}=2\vec{a}\cdot \vec{r_{1}}-\vec{a}$$

(24) $$\vec{C}=2\cdot \vec{r_{2}}$$

where $\vec{r_{1}}$ and $\vec{r_{2}}$ are random vectors between [0, 1], and $\vec{a}$ is the convergence factor, which linearly decreases from the form of Equation (25) to 0 as the number of iterations increases.

(25) $$\vec{a}=2-\frac{2t}{t_{\max}}$$

where $t_{\max}$ is the maximum number of iterations. When A>1, the whales will randomly search for food $\vec{X_{rand}}$, that is, individual whales will randomly search each other based on their location.

The position (X,Y) of a searching individual can be updated to the current optimal position $\left(X^{*},Y^{*}\right)$ according to Equation (22) and the optimal position can be reached at different positions around the best individual by adjusting the vector values of $\vec{A}$ and $\vec{C}$. There are two different approaches to the bubble net feeding behavior of whales, the constriction encircling mechanism and the spiral replacement position. Thus, the mathematical model of whale bubble feeding behavior is as follows:

(26) $$\vec{X}(t+1)=\left\{\begin{array}{lll} \vec{X^{*}}(t)-\vec{A}\cdot \vec{D} & , & p<0.5\\ \vec{D^{’}}\cdot e^{bl}\cdot \cos(2\pi l)+\vec{X^{*}}(t) & , & p\geq 0.5 \end{array}\right.$$

where $\vec{D^{’}}$ is the distance between the i-th whale and the prey, b is the constant used to define the logarithmic spiral shape, and l is the random number between $\left[-1,1\right]$. In the optimization process, the selection of the enveloping mechanism and spiral position update probability p are both 0.5.

**Figure 1:**
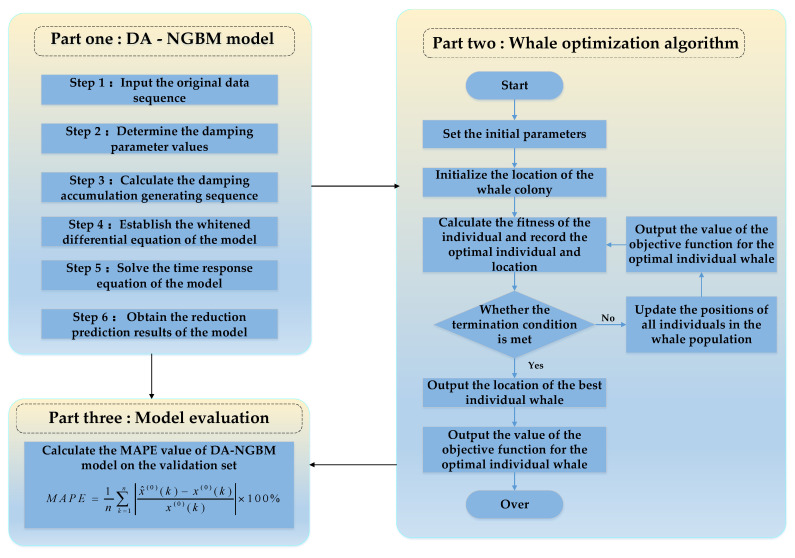
Modeling flow chart of DA–NGBM model.

The modeling flow chart of the DA–NGBM model is shown in Figure 1.

### 3.3. Research on the Properties of the Model

The DA–NGBM model has the following two basic properties:

**Property** **4.** *When* ζ=1*, the damped accumulation operator degenerates into the traditional first-order accumulation operator and the DA–NGBM model degenerates into the NGBM model.*

**Property** **5.** *When* ζ=1*,* α=0*, the DA–NGBM model degenerates into the traditional GM(1,1) model.*

#### 3.3.1. Reduction Error Analysis

**Theorem** **1.** *Suppose that the non-negative sequence of the original data is* $X^{\left(0\right)}=\{x^{\left(0\right)}(1),x^{\left(0\right)}(2),\cdots ,x^{\left(0\right)}(n)\}$ *and the corresponding damped accumulation generation sequence of the original data is* $X^{\left(\zeta \right)}=\{x^{\left(\zeta \right)}(1),x^{\left(\zeta \right)}(2),\cdots ,x^{\left(\zeta \right)}(n)\}$*. The response value sequence of the DA–NGBM model is* ${\hat{X}}^{\left(\zeta \right)}=\{{\hat{x}}^{\left(\zeta \right)}(1),{\hat{x}}^{\left(\zeta \right)}(2),\cdots ,{\hat{x}}^{\left(\zeta \right)}(n)\}$ *and the prediction sequence of the model is* ${\hat{X}}^{\left(0\right)}=\{{\hat{x}}^{\left(0\right)}(1),{\hat{x}}^{\left(0\right)}(2),\cdots ,{\hat{x}}^{\left(0\right)}(n)\}$*. If the reduction error of the response value of the DA–NGBM model meets* $\left|{\hat{x}}^{\left(\zeta \right)}(k)-x^{\left(\zeta \right)}(k)\right|\leq \varepsilon$*, then the reduction error of the model meets* $\left|{\hat{x}}^{\left(0\right)}(k)-x^{\left(0\right)}(k)\right|\leq 2\zeta^{k-1}\varepsilon$.

**Proof** **of** **Theorem** **1.** It can be seen from the modeling results of the DA–NGBM model that

$$({\hat{x}}^{\left(0\right)}(1),{\hat{x}}^{\left(0\right)}(2),\cdots ,{\hat{x}}^{\left(0\right)}(n))=({\hat{x}}^{\left(\zeta \right)}(1),{\hat{x}}^{\left(\zeta \right)}(2),\cdots ,{\hat{x}}^{\left(\zeta \right)}(n)){\left(\begin{array}{cccc} 1 & 1 & \cdots & 1\\ 0 & \frac{1}{\zeta } & \cdots & 1\\ \vdots & \vdots & \cdots & \vdots \\ 0 & 0 & \cdots & \frac{1}{\zeta^{n-1}} \end{array}\right)}^{-1},$$

Then, the reduction value of the model can be obtained by reduction as ${\hat{x}}^{\left(0\right)}(k)=({\hat{x}}^{\left(\zeta \right)}(k)-{\hat{x}}^{\left(\zeta \right)}(k-1))\zeta^{k-1}$.

Since it is known that the reduction error of the response value of the DA–NGBM model meets $\left|{\hat{x}}^{\left(\zeta \right)}(k)-x^{\left(\zeta \right)}(k)\right|<\varepsilon$, then

$$\begin{array}{cc} \left|{\hat{x}}^{\left(0\right)}(k)-x^{\left(0\right)}(k)\right| & =\left|({\hat{x}}^{\left(\zeta \right)}(k)-{\hat{x}}^{\left(\zeta \right)}(k-1))\zeta^{k-1}-(x^{\left(\zeta \right)}(k)-x^{\left(\zeta \right)}(k-1))\zeta^{k-1}\right|\\ & =\left|({\hat{x}}^{\left(\zeta \right)}(k)-x^{\left(\zeta \right)}(k))\zeta^{k-1}-({\hat{x}}^{\left(\zeta \right)}(k-1)-x^{\left(\zeta \right)}(k-1))\zeta^{k-1}\right|\\ & \leq \left|({\hat{x}}^{\left(\zeta \right)}(k)-x^{\left(\zeta \right)}(k))\zeta^{k-1}\right|+\left|({\hat{x}}^{\left(\zeta \right)}(k-1)-x^{\left(\zeta \right)}(k-1))\zeta^{k-1}\right|\\ & \leq 2\zeta^{k-1}\varepsilon \end{array}$$

That is, when the reduction error of the response value of the DA–NGBM model is $\left|{\hat{x}}^{\left(\zeta \right)}(k)-x^{\left(\zeta \right)}(k)\right|\leq \varepsilon$, the reduction error of the model is $\left|{\hat{x}}^{\left(0\right)}(k)-x^{\left(0\right)}(k)\right|\leq 2\zeta^{k-1}\varepsilon$. □

**Property** **6.** *The upper limit of reduction error of the predicted value of the DA–NGBM model is positively correlated with the damping parameter*ζ*and negatively correlated with the time variable* k.

#### 3.3.2. Analysis of Prediction Results

**Property** **7.** *The exponential growth rate of the predicted results of the DA–NGBM model is positively correlated with the value of the damping parameter*ζ.

**Proof** **of** **Property** **7.** 
$$\begin{array}{cc} {\hat{x}}^{\left(0\right)}\left(k+1\right) & =\left({\hat{x}}^{\left(\zeta \right)}\left(k+1\right)-{\hat{x}}^{\left(\zeta \right)}\left(k\right)\right)\zeta^{k}\\ & =\left\{{\left[\frac{b}{a}+\left({\left(x^{\left(0\right)}(1)\right)}^{1-\alpha }-\frac{b}{a}\right)e^{-\left(1-\alpha \right)ak}\right]}^{\frac{1}{1-a}}-{\left[\frac{b}{a}+\left({\left(x^{\left(0\right)}(1)\right)}^{1-\alpha }-\frac{b}{a}\right)e^{-\left(1-\alpha \right)a\left(k-1\right)}\right]}^{\frac{1}{1-a}}\right\}\cdot \zeta^{k} \end{array}$$

Considering the complexity of simplified calculation, the detailed proof of the determined parameters is ignored. Then, the above equation can be simplified as

$$\begin{array}{l} {\hat{x}}^{\left(0\right)}\left(k+1\right)=\left\{{\left[\frac{b}{a}+c\cdot e^{-\left(1-\alpha \right)ak}\right]}^{n}-{\left[\frac{b}{a}+c\cdot e^{-\left(1-\alpha \right)a\left(k-1\right)}\right]}^{n}\right\}\cdot \zeta^{k}\\ =\left\{\left[C_{n}^{0}\cdot {\left(c\cdot e^{-\left(1-\alpha \right)ak}\right)}^{0}\cdot {\left(\frac{b}{a}\right)}^{n}+C_{n}^{1}\cdot {\left(c\cdot e^{-\left(1-\alpha \right)ak}\right)}^{1}\cdot {\left(\frac{b}{a}\right)}^{n-1}+\cdot \cdot \cdot +C_{n}^{n}\cdot {\left(c\cdot e^{-\left(1-\alpha \right)ak}\right)}^{n}\cdot {\left(\frac{b}{a}\right)}^{0}\right]\right.-\\ \;\left.\left[C_{n}^{0}\cdot {\left(c\cdot e^{-\left(1-\alpha \right)a\left(k-1\right)}\right)}^{0}\cdot {\left(\frac{b}{a}\right)}^{n}+C_{n}^{1}\cdot {\left(c\cdot e^{-\left(1-\alpha \right)a\left(k-1\right)}\right)}^{1}\cdot {\left(\frac{b}{a}\right)}^{n-1}+\cdot \cdot \cdot +C_{n}^{n}\cdot {\left(c\cdot e^{-\left(1-\alpha \right)a\left(k-1\right)}\right)}^{n}\cdot {\left(\frac{b}{a}\right)}^{0}\right]\right\}\cdot \zeta^{k}\;\\ =\left\{C_{n}^{0}\cdot c^{0}\cdot {\left(\frac{b}{a}\right)}^{n}\cdot {\left[e^{-\left(1-\alpha \right)ak}\left(1-e^{\left(1-\alpha \right)a}\right)\right]}^{0}+C_{n}^{1}\cdot c^{1}\cdot {\left(\frac{b}{a}\right)}^{n-1}\cdot {\left[e^{-\left(1-\alpha \right)ak}\left(1-e^{\left(1-\alpha \right)a}\right)\right]}^{1}+\right.\cdots +\left.C_{n}^{n}\cdot c^{n}\cdot {\left(\frac{b}{a}\right)}^{0}\cdot {\left[e^{-\left(1-\alpha \right)ak}\left(1-e^{\left(1-\alpha \right)a}\right)\right]}^{n}\right\}\cdot \zeta^{k}\\ =\left\{C_{0}\cdot {\left[e^{-\left(1-\alpha \right)a\cdot 0}\right]}^{k}+C_{1}\cdot {\left[e^{-\left(1-\alpha \right)a\cdot 1}\right]}^{k}+\cdots +C_{n}\cdot {\left[e^{-\left(1-\alpha \right)a\cdot n}\right]}^{k}\right\}\cdot \zeta^{k}\\ =C_{0}\cdot {\left[e^{-\left(1-\alpha \right)a\cdot 0}\cdot \zeta \right]}^{k}+C_{1}\cdot {\left[e^{-\left(1-\alpha \right)a\cdot 1}\cdot \zeta \right]}^{k}+\cdots +C_{n}\cdot {\left[e^{-\left(1-\alpha \right)a\cdot n}\cdot \zeta \right]}^{k} \end{array}$$

where $C_{i}=C_{n}^{i}\cdot c^{i}\cdot {\left(\frac{b}{a}\right)}^{n-i}\cdot {\left(1-e^{\left(1-\alpha \right)a}\right)}^{i},\left(i=1,2,\cdots ,n\right)$, $c=\left({\left(x^{\left(0\right)}(1)\right)}^{1-\alpha }-\frac{b}{a}\right)$, $n=\frac{1}{1-\alpha }$.

Consider the above equation as a function of the time variable, equivalent to $f\left(t+1\right)=\sum_{i=1}^{n}C_{i}\cdot \eta^{t}$. It can be seen that the prediction results of the DA–NGBM model still have exponential growth form. According to the properties of the exponential function, the growth rate of model prediction results is controlled by the base number η. The larger η is, the larger the value of the damping parameter ζ is and the larger the exponential growth rate of the model prediction results is. □

#### 3.3.3. Disturbance Bound Analysis

**Lemma** **1**  **([24]).** $A\in C^{m\times n}$*,* $b\in C^{m}$*. Suppose* $A^{†}$ *is the generalized inverse of the matrix* A*,* B=A+E*,* $c=b+k\in C^{m}$ *. Suppose* x+h *and* x *are solutions of the linear least squares problem* ${\left\|Bx-c\right\|}_{2}=\min$ *and* ${\left\|Ax-b\right\|}_{2}=\min$ *, respectively. If* rank(A)=rank(B)=n *and* ${\left\|A^{†}\right\|}_{2}{\left\|E\right\|}_{2}<1$ *, then we have*

(27) $$\left\|h\right\|\leq \frac{\kappa_{†}}{\gamma_{†}}\left(\frac{{\left\|E\right\|}_{2}}{\left\|A\right\|}\left\|x\right\|+\frac{\left\|k\right\|}{\left\|A\right\|}+\frac{\kappa_{†}}{\gamma_{†}}\frac{{\left\|E\right\|}_{2}}{\left\|A\right\|}\frac{\left\|r_{x}\right\|}{\left\|A\right\|}\right)$$

*where* $\kappa_{†}={\left\|A^{†}\right\|}_{2}{\left\|A\right\|}_{2}$*,*  $\gamma_{†}=1-{\left\|A^{†}\right\|}_{2}{\left\|E\right\|}_{2}$*,*  $r_{x}=b-A_{x}$.

**Theorem** **2.** *Suppose* x *is the solution of the DA–NGBM model satisfying the least square method* $\min{\left\|Bx-Y\right\|}_{2}$ *. If* $\varepsilon =\left|\Delta x\right|$ *is the perturbation value of the initial sequence data* $x^{\left(0\right)}(i),\;(i=1,2,\cdot \cdot \cdot ,n)$ *, the perturbation boundary of the solution of the model* x *increases with the increase in the data volume and decreases with the increase in the damping parameters.*

**Proof** **of** **Theorem** **2.** When only $x^{\left(0\right)}(1)$ disturbs ε, put ${\hat{x}}^{\left(0\right)}(1)=x^{\left(0\right)}(1)+\varepsilon$ into Equation (11) and we can obtain

$$\hat{B}+\Delta B=\left[\begin{array}{cc} -z^{\left(\zeta \right)}\left(2\right) & {\left(z^{\left(\zeta \right)}\left(2\right)\right)}^{\alpha }\\ -z^{\left(\zeta \right)}\left(3\right) & {\left(z^{\left(\zeta \right)}\left(3\right)\right)}^{\alpha }\\ \vdots & \vdots \\ -z^{\left(\zeta \right)}\left(3\right) & {\left(z^{\left(\zeta \right)}\left(n\right)\right)}^{\alpha } \end{array}\right]+\left[\begin{array}{cc} \begin{array}{c} -\varepsilon \\ -\varepsilon \\ \vdots \\ -\varepsilon \end{array} & \begin{array}{c} \varepsilon^{\alpha }\\ \varepsilon^{\alpha }\\ \vdots \\ \varepsilon^{\alpha } \end{array} \end{array}\right],\;\hat{Y}+\Delta Y=\left[\begin{array}{c} x^{\left(\zeta \right)}(2)-x^{\left(\zeta \right)}(1)\\ x^{\left(\zeta \right)}(3)-x^{\left(\zeta \right)}(2)\\ \vdots \\ x^{\left(\zeta \right)}(n)-x^{\left(\zeta \right)}(n-1) \end{array}\right]+\left[\begin{array}{c} 0\\ 0\\ \vdots \\ 0 \end{array}\right].$$

Therefore, ${\left\|\Delta Y\right\|}_{2}=0$, $\Delta B^{T}\Delta B=\left[\begin{array}{cc} \begin{array}{c} \sum_{i=1}^{n-1}\varepsilon^{2}\\ -\sum_{i=1}^{n-1}\varepsilon^{\alpha +1} \end{array} & \begin{array}{c} -\sum_{i=1}^{n-1}\varepsilon^{\alpha +1}\\ \sum_{i=1}^{n-1}\varepsilon^{2\alpha } \end{array} \end{array}\right]$, ${\left\|\Delta B\right\|}_{2}=\sqrt{\left(n-1\right)\varepsilon^{2\alpha }+\left(n-1\right)\varepsilon^{2}}$, according to Lemma 1, the disturbance bound of the model solution is

$$L\left[x^{\left(0\right)}(1)\right]=\frac{\kappa_{†}}{\gamma_{†}}\sqrt{\left(n-1\right)\varepsilon^{2\alpha }+\left(n-1\right)\varepsilon^{2}}\left(\frac{1}{\left\|B\right\|}\left\|x\right\|+\frac{\kappa_{†}}{\gamma_{†}}\frac{1}{\left\|B\right\|}\frac{\left\|r_{x}\right\|}{\left\|B\right\|}\right).$$

When only $x^{\left(0\right)}(i)$(i=2,3,⋅⋅⋅,n−1) is disturbed ε,

$${\left\|\Delta Y\right\|}_{2}=\frac{\varepsilon }{\zeta^{i-1}},\;{\left\|\Delta B\right\|}_{2}=\sqrt{\frac{\left(\Delta B_{11}+\Delta B_{22}\right)+\sqrt{{\left(\Delta B_{11}+\Delta B_{22}\right)}^{2}-4\cdot (n-i)\cdot \left(\frac{1}{2^{2\alpha }}+\frac{1}{4}-\frac{1}{2^{\alpha }}\right)\cdot {\left(\frac{\varepsilon }{\zeta^{i-1}}\right)}^{2\alpha +2}}}{2}},$$

where $\Delta B_{11}={\left(\frac{1}{2}\cdot \frac{\varepsilon }{\zeta^{i-1}}\right)}^{2}+\left(n-i\right){\left(\frac{\varepsilon }{\zeta^{i-1}}\right)}^{2}$, $\Delta B_{22}={\left(\frac{1}{2}\cdot \frac{\varepsilon }{\zeta^{i-1}}\right)}^{2\alpha }+\left(n-i\right){\left(\frac{\varepsilon }{\zeta^{i-1}}\right)}^{2\alpha }$.

By substituting the above results into Equation (27), the disturbance bound of the model solution can be obtained as

$$L\left[x^{\left(0\right)}(i)\right]=\frac{\kappa_{†}}{\gamma_{†}}\left(\frac{{\left\|\Delta B\right\|}_{2}}{\left\|B\right\|}\left\|x\right\|+\frac{1}{\left\|B\right\|}\cdot \frac{\varepsilon }{\zeta^{i-1}}+\frac{\kappa_{†}}{\gamma_{†}}\frac{{\left\|\Delta B\right\|}_{2}}{\left\|B\right\|}\frac{\left\|r_{x}\right\|}{\left\|B\right\|}\right)\propto \sqrt{n}$$

Observe that only $x^{\left(0\right)}(n)$ is disturbed ε, ${\hat{x}}^{\left(0\right)}(n)=x^{\left(0\right)}(n)+\varepsilon$,

$${\left\|\Delta Y\right\|}_{2}=\frac{\varepsilon }{\zeta^{n-1}},\;\Delta B^{T}\Delta B=\left[\begin{array}{cc} \begin{array}{c} \frac{1}{4}{\left(\frac{\varepsilon }{\zeta^{n-1}}\right)}^{2}\\ -{\left(\frac{1}{2}\cdot \frac{\varepsilon }{\zeta^{n-1}}\right)}^{\alpha +1} \end{array} & \begin{array}{c} -{\left(\frac{1}{2}\cdot \frac{\varepsilon }{\zeta^{n-1}}\right)}^{\alpha +1}\\ {\left(\frac{1}{2}\cdot \frac{\varepsilon }{\zeta^{n-1}}\right)}^{2\alpha } \end{array} \end{array}\right],\;{\left\|\Delta B\right\|}_{2}=\sqrt{{\left(\frac{1}{2}\cdot \frac{\varepsilon }{\zeta^{n-1}}\right)}^{2}+{\left(\frac{1}{2}\cdot \frac{\varepsilon }{\zeta^{n-1}}\right)}^{2\alpha }}.$$

According to Lemma 1, the disturbance bound of the model solution is

$$L\left[x^{\left(0\right)}(n)\right]=\frac{\kappa_{†}}{\gamma_{†}}\left(\frac{1}{\left\|B\right\|}\sqrt{{\left(\frac{1}{2}\cdot \frac{\varepsilon }{\zeta^{n-1}}\right)}^{2}+{\left(\frac{1}{2}\cdot \frac{\varepsilon }{\zeta^{n-1}}\right)}^{2\alpha }}\left\|x\right\|+\frac{1}{\left\|B\right\|}\cdot \frac{\varepsilon }{\zeta^{n-1}}+\frac{\kappa_{†}}{\gamma_{†}}\frac{1}{\left\|B\right\|}\sqrt{{\left(\frac{1}{2}\cdot \frac{\varepsilon }{\zeta^{n-1}}\right)}^{2}+{\left(\frac{1}{2}\cdot \frac{\varepsilon }{\zeta^{n-1}}\right)}^{2\alpha }}\frac{\left\|r_{x}\right\|}{\left\|B\right\|}\right),$$

which conforms to the conclusion deduced above. To sum up, the disturbance bound of the solution of the model x increases with the increase in data quantity and decreases with the increase in the damping parameter value. □

The matrix perturbation boundary theory proves that the essence of the grey prediction model of the DA–NGBM model remains unchanged despite the optimization of the original model. As a prediction model suitable for “small sample” data, the disturbance boundary of the DA–NGBM model increases with the increase in data sample size. To keep the information updated constantly and remove the old information in time, it is convenient to avoid the large disturbance of the predicted value of the model and also highlights that the new information has more important guiding significance for the prediction model. The selection of damping parameter value plays an important role in the stability of the predicted value of the model. Therefore, the influence of the change of damping parameter value on the prediction accuracy of the model should be fully considered in the prediction process.

### 3.4. Example Analysis

Based on the above research on the properties of the DA–NGBM model and the method of parameter selection, the following example is used to make a practical comparison between DA–NGBM and the classical population model—the Logistics model [36]. The number of the registered population in Shanghai from 2009 to 2018 is selected as the basic data. Through data fitting, the fitting value of the registered population in Shanghai from 2011 to 2018 is obtained and the number of registered population in Shanghai from 2019 to 2020 is predicted. The fitting error and prediction error are shown in Table 2. By calculating the MAPE value of the two prediction models, it can be found that the fitting accuracy and prediction accuracy of the DA–NGBM model were greatly improved compared with the logistic competitive biological model.

## 4. Case Analysis of Population Prediction

The proposed DA–NGBM model provides a new idea for solving practical problems by using the grey prediction model. The behavior sequence of the model is constructed by using the damping accumulated method, which can not only satisfy the “new information first” criterion in grey modeling but also enhance the prediction accuracy and stability of the model to a certain extent. In order to verify the prediction effect of the DA–NGBM model after changing the accumulative method, three numerical calculation cases related to the prediction of population size and structure in China are selected in this section. Compared with the NGBM model, the original GM(1,1) model, the DA–GM (1,1) model [34], the deep learning model–LSTM [37] and the traditional statistical model–Exponential smoothing, the prediction ability of the improved DA–NGBM model was analyzed and evaluated.

**Figure 2:**
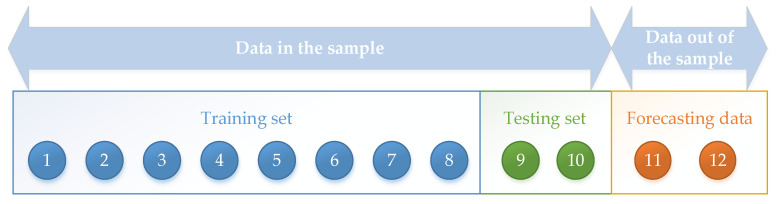
Division of sample data.

The three cases selected in this paper contain ten groups of data and the first eight groups of data in the sample are used as the training set for the model. First, the eight data are used to fit the parameters of the model through the whale optimization algorithm. The whale optimization algorithm is used to find the optimal power exponent and the optimal damping parameters to minimize the fitting error of the model, and the optimal solution can be found by setting the searching times to 50 times. The last two groups of data in the sample are taken as the testing set for the model. On the premise of obtaining model parameters, the DA–NGBM model and the traditional grey prediction model are used for two-step prediction. The error analysis is made between the obtained prediction results and the data in the testing set to compare the prediction ability of the model. The specific division of sample data is shown in Figure 2.

### 4.1. China’s Birth Rate Forecast from 2011 to 2020

According to the birth rate data of China from 2011 to 2020 in the China Statistical Yearbook 2021 [38], the DA–NGBM model and traditional NGBM model, traditional GM(1,1) model, DA–GM(1,1) model, LSTM and Exponential smoothing are used to conduct comparative experiments to fit and predict the birth rate of the Chinese population, respectively. The birth rate from 2011 to 2018 was used to fit the model, the birth rate from 2019 to 2020 was used as the testing set, and the average absolute error percentage (MAPE) was used as the absolute standard to judge the quality of the prediction model and then the prediction accuracy of the four grey prediction models, one deep learning model and one traditional statistical model, was compared. It can be found that after adding the damping accumulated factor and intelligent optimization algorithm, the fitting and prediction accuracy of the DA–NGBM model is still greatly improved from the original model when the predicted data has obvious floating.

The comparison and prediction results of the six models are shown in Table 3. It can be found that the LSTM prediction model has the highest fitting accuracy after 5000 times of training. In terms of prediction, since this is a small sample problem, the LSTM model lacks sufficient training samples, therefore the prediction effect of it is not so good. To some extent, deep learning algorithms that require large sample data are not suitable for the prediction of small sample problems. The average fitting error of the DA–NGBM model is 4.63%, the prediction error for 2019 is 2.40%, and the prediction error for 2020 is 17.96%. China’s birth rate fluctuated due to the COVID-19 outbreak in 2020. However, under such data conditions with obvious fluctuations, the prediction effect of the DA–NGBM model is still optimal. It can be proved that by introducing the damping accumulated factor into the NGBM model, the prediction ability of the model is further improved and that this method is an effective improvement.

In this example, the optimal power index and damping parameter values of the DA–NGBM α=0.0975, ζ=0.7359 are calculated by MATLAB software and the model parameters obtained by least square are a=−0.2446, b=10.5439, respectively.

### 4.2. Prediction of Registered Population in Jiangsu Province from 2011 to 2020

According to the data of the registered total population in Jiangsu Province from 2011 to 2020 in the Jiangsu Statistical Yearbook 2021 [39], six kinds of prediction models are used for prediction, the total registered population of Jiangsu from 2011 to 2018 is taken as the fitting data, and the total registered population of Jiangsu from 2019 to 2020 is taken as the testing data. The comparison and prediction results of the six models are shown in Table 4. It can be seen that the total registered population in Jiangsu is a kind of data that has an overall trend of monotonically increasing. In the fitting calculation of this type of data series, the average fitting error of the DA–NGBM model is the first among six types of prediction models with a small error of 0.06%, which is further improved compared with the original NGBM’s average fitting error of 0.08%.

For the prediction data, compared with the prediction results of 2019, the prediction error of the original NGBM model is 0.14%, the prediction error of the original GM(1,1) model is 0.47%, and the prediction error of the DA–GM(1,1) model is about 0.2% lower than the original model. The DA–NGBM model still ranks first among the four models with a prediction error of only 0.1%. Similarly, the prediction data of 2020 were observed and the prediction error of the DA–NGBM model was 0.29% as the optimal prediction model. The performance of the deep learning model and the traditional statistical method in this case also has a certain gap with that of the new grey forecasting method. Therefore, in this kind of monotonically increasing data prediction, the prediction ability of the DA–NGBM should be affirmed.

In this case, the optimal power index and damping parameter values of the DA–NGBM model α=0.0114, ζ=0.9458 are calculated by MATLAB software and the model parameters obtained by least square are a=−0.0590, b=6573.73, respectively.

### 4.3. Prediction of the Male Population of Nanjing City from 2011 to 2020

According to the data of the male population of Nanjing city from 2011 to 2020 in the Nanjing Statistical Yearbook 2021 [40], four kinds of grey prediction models, a deep learning model and a traditional statistical method, are used for prediction. The total male population of Nanjing city from 2011 to 2018 is taken as the fitting data and the male population of Nanjing city from 2019 to 2020 is taken as the testing data. The comparison and prediction results of the six models are shown in Table 5. It can be seen that the total male population of Nanjing city is a kind of data with an overall trend that is monotonically increasing.

In the fitting calculation of this type of data series, the fitting effect of the LSTM method is the most accurate. However, the prediction effect of the LSTM model is worse than the DA–NGBM model in the testing stage and the prediction results of the DA–NGBM for two years are closest to the real value, with prediction errors of only 0.36% and 0.09%, which has the best prediction ability among the six predicted models. By comparison, the grey method shows superior ability in solving small sample prediction problems. It should be noted that the GM(1,1) model and the DA–GM(1,1) model obtained the same result in this case because the optimal value of the damping parameter of the DA–GM(1,1) model was selected as 1, therefore the DA–GM (1,1) model degenerated into the classical GM(1,1) model.

In this example, the optimal power index and damping parameter values of the DA–NGBM α=−0.0468, ζ=0.9677 are calculated by MATLAB software and the model parameters obtained by least square are a=−0.0558, b=407.1498, respectively.

## 5. Discussion

**Figure 3:**
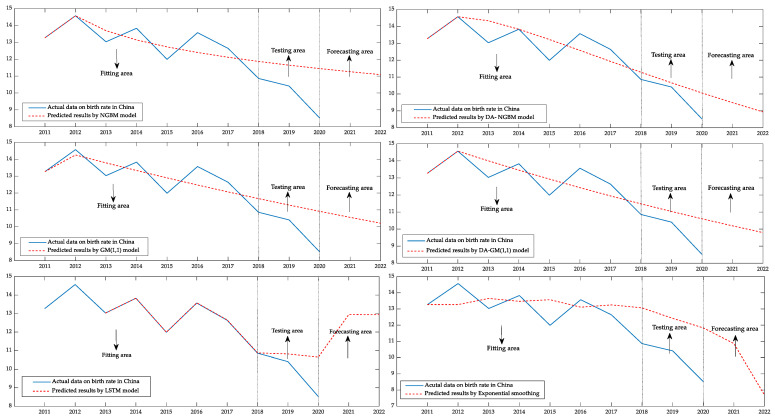
Comparison of the fitting and forecast curves of China’s birth rate from 2011 to 2022.

In order to show the fitting and prediction ability of the DA–NGBM model more vividly, this example is divided into two experimental areas. The birth rate of China from 2011 to 2018 is regarded as the fitting data and the birth rate from 2019 to 2020 is regarded as the testing data. The birth rate of China in 2021–2022 predicted by the six types of comparison models in this example is regarded as the forecasting data, and the fitting, testing and forecasting results of the six types of models are visualized by the MATLAB program, as shown in Figure 3.

The data, in this case, fluctuated greatly and showed a downward trend as a whole. It can be found that the GM(1,1) model and the NGBM model, as traditional grey prediction methods, can only match the real value at a few points and that these models do not have a strong perception ability for the changing trend of a data series. The optimized GM(1,1) model has a certain improvement in data fitting but there is still a certain deviation from the real value in the test results. The performance of the traditional statistical method, in this case, is even less than satisfactory. Their prediction results are difficult to correctly correspond with population changes, the description of the changing trend is not clear, and there are large errors in the fitting and testing stages, therefore the accuracy of future prediction results may not be too high. From the results curve of the LSTM model, it can be found that in the fitting area, the curve of the LSTM model is almost exactly consistent with the real data. However, in the testing area, the prediction curve of the LSTM model is significantly different from the real value and shows an opposite trend of change. Therefore, it can be confirmed that the LSTM model still has excellent fitting ability in small sample problems but it is not completely suitable for the prediction problem of small sample cases.

It is not difficult to find that the DA–NGBM model performs better than the other three grey models in fitting and predicting the original behavior sequences with large fluctuations. It can also be seen that this model has good stability and will not affect the overall prediction result because of the anomaly of a single point. Not only in the fitting stage is the data curve smooth and conforms to the law of population growth, which is suitable for the prediction of population system, but also in the prediction process, the multi-point data can be consistent with the real value, and also in the test results, it is close to the real trend of population change. 

**Figure 4:**
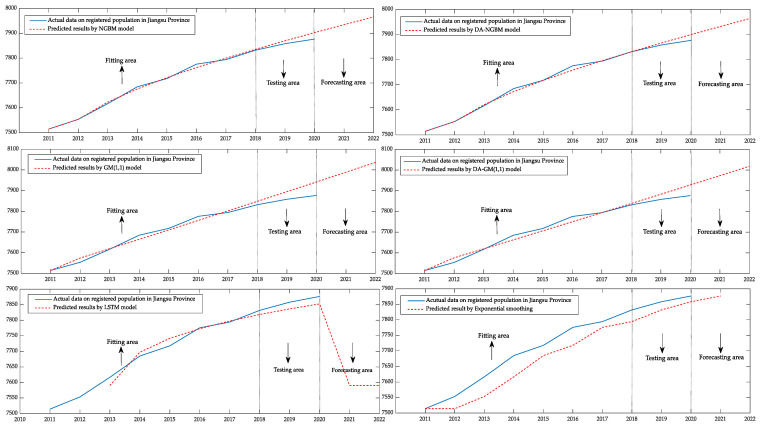
Comparison of the fitting and prediction curves of registered population in Jiangsu Province from 2011 to 2022.

The registered population of Jiangsu province from 2011 to 2018 is the fitting data and the registered population of Jiangsu Province from 2019 to 2020 is taken as the testing data. The registered population of Jiangsu Province from 2021 to 2022 is taken as the forecasting data of the six prediction models. The fitting, testing and forecasting results of the six types of models are visualized through the MATLAB program. The result is shown in Figure 4.

The data fluctuation of this case is small and shows an overall upward trend. It can be found that the fitting and test coincidence of several types of grey models are quite ideal cause the original sequence changes regularly. The traditional statistical method can also describe the changing trend of the population but there is a large deviation from the actual value. The LSTM model can show more accurate results in the fitting and testing area but the predicted value in the prediction area has changed significantly and the change mode does not conform to the law of population change in reality. Although the results obtained by the four types of grey prediction models are satisfactory, careful observation shows that, compared with the GM(1,1) model, the NGBM model with nonlinear characteristics has better performance in grasping data changes. It is more consistent with the real data in the fitting stage and has significantly less errors in the test stage than the GM(1,1) model. The deviation between the predicted curve and the real curve is small. This also reflects that the linear model has certain disadvantages. In order to make use of the advantages of the nonlinear model in mastering data changes, this paper has optimized the NGBM model.

After the improvement of the original model, the prediction curve of the DA–NGBM model coincides highly with the actual data curve in the fitting stage. Compared with the DA–GM (1,1), the NGBM model, LSTM and Exponential smoothing, the DA–NGBM model has obvious improvement and the best fitting ability. In the testing stage, the deviation between the predicted results and the real data is the smallest among the six models. This shows that the DA–NGBM model has high prediction accuracy and the optimization in the NGBM model has a good effect.

**Figure 5:**
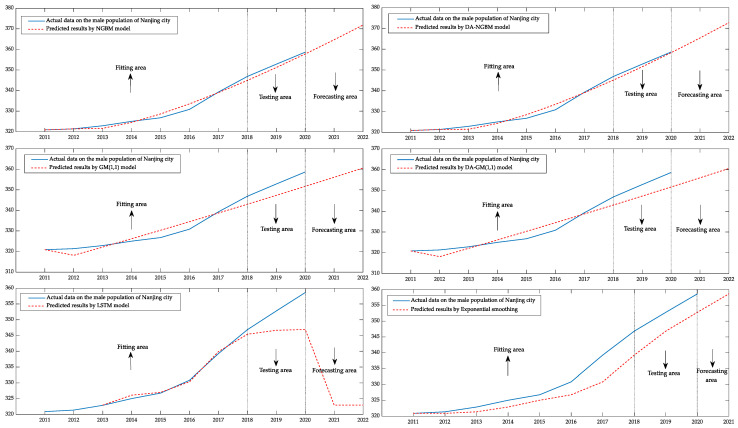
Comparison of the fitting and prediction curves of the male population of Nanjing city from 2011 to 2022.

Next, we took the male population of Nanjing city from 2011 to 2018 as the fitting data and the male population of Nanjing city from 2019 to 2020 as the testing data. The male population of Nanjing city from 2021 to 2022 is taken as the forecasting data of the four types of grey prediction models, LSTM and Exponential smoothing. The fitting, testing and forecasting results of the six types of models are visualized through the MATLAB program. The result is shown in Figure 5.

Through the six images, in this case, the superior fitting and prediction ability of the DA–NGBM model can be demonstrated more clearly. It can be seen that although the NGBM model without optimization also has a good fitting ability, the prediction result of the DA–NGBM model is closer to the real value curve in the test stage. The predicted curves of the GM(1,1) model and the improved GM(1,1) model were quite different from the real value, especially in the testing phase, and could not accurately describe the changing trend of this population data. The LSTM model shows superior ability in the fitting region, but compared with the grey prediction model, the prediction deviation of the LSTM and the traditional statistical models is higher than the DA–NGBM and the results have less reliability.

Based on the above three cases, compared with the other five comparison models, the DA–NGBM model can deeply explore the fluctuation form of the population system and closely capture the overall evolution trend and individual change of this dynamic system, no matter if the original data have obvious fluctuation characteristics or the data series have monotonous growth (decline) trends. It is more reliable in fitting and prediction accuracy, especially in its prediction effect. This work, therefore, has great practical significance in population prediction. Promoting a country’s effective grasp of the trend of population change can provide guiding opinions for the country to formulate relevant population policies and play an important role in strengthening social governance, therefore this issue is suitable for further study and application in population system prediction analysis.

**Figure 6:**
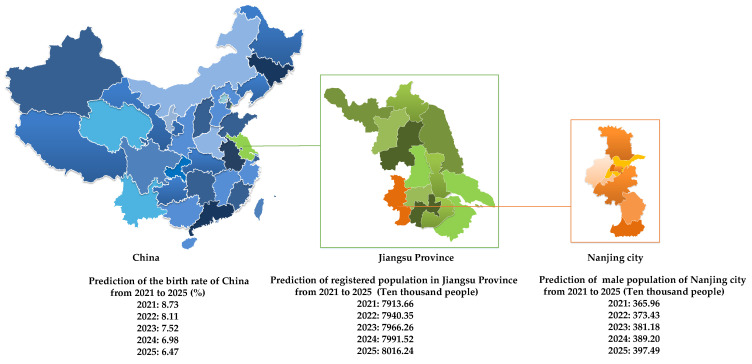
Projections of the size and structure of China’s population from 2021 to 2025.

After verifying the effectiveness of the DA–NGBM model, the DA–NGBM was used to predict the birth rate of China, the number of registered residents in Jiangsu Province, and the male population of Nanjing city from 2021 to 2015. The prediction results are shown in Figure 6.

## 6. Conclusions and Future Work

Correctly understanding and handling the relationship between population development and resources, environment and economy, and implementing the strategy of sustainable development, which is a major issue related to the rise and fall of a country and the survival of a nation, is also the eternal theme of mankind. As a direct means of adjusting and controlling population indicators, population decision-making plays an important role in economic and social development, and the accurate prediction of the population is directly related to the scientific decision-making of population issues. Therefore, selecting a reasonable and effective population prediction model, grasping the size of the population accurately, and understanding its development situation, have far-reaching significance for the formulation of the national economic plan and social development strategy.

### 6.1. Conclusions

Taking into account the importance attached to population prediction by countries around the world, the need for population prediction in formulating population policies and social development strategies, and the improvement of social welfare facilities in order to promote sustainable development of society, and in order to promote the sustainable development of society, perfect social welfare facilities, a kind of grey Bernoulli model with damping accumulation is established in this paper. As an optimization of the traditional grey prediction model, this model has an obvious improvement effect on the reasonable prediction of population system change. The NGBM model is a nonlinear grey model essentially, whose flexible power index enables it to have a broader ability to solve nonlinear feature problems and a strong universality compared with the traditional GM(1,1) model. In this paper, the damping accumulated operator is integrated into the data sequence construction of the NGBM model, and the intelligent advantage of the whale optimization algorithm is used to find the parameter value of the model in order to make the prediction model more sensitive to the changes of the original data and enhance the accuracy and stability of the prediction model, as well as reduce the complexity of calculating and use convenient research.

Combined with three cases of birth rate in China, the total registered population in Jiangsu Province and the male population of Nanjing city, this paper studies the DA–NGBM model compared with three kinds of grey prediction methods and two traditional statistical methods. The result shows that the new prediction model can fully adapt to the original series with different development trend characteristics and has the best fitting and prediction performance. The experimental results show that the predicted curves obtained by the DA–NGBM model are in better agreement with the real data curves in both the fitting and testing stages. At the same time, the addition of the damping accumulated operator can effectively adjust the exponential growth rate of the predicted results and also ensure the reliability and stability of the predicted results for the fluctuating original data series. As a result, the DA–NGBM model can effectively predict future population changes reliably. Therefore it is believed that the DA–NGBM model will have a broad research background and application space in population prediction and it is worth promoting to the application research of relevant population problems.

### 6.2. Future Work

As an important part of grey system theory, the grey prediction model reduces the randomness of the original sequence by cumulation operator and then introduces the data into the construction equation with biased and partial differential characteristics. Through this method, the inherent law of data series is fully mined, scientific and quantitative prediction is made, and the future development trend of the system is revealed. Grey system theory is a new system science method first proposed by Julong Deng in 1982 and has only been developed for 40 years. Therefore, some theories in this discipline are still not mature and many valuable theories have not yet been deeply explored. The DA–NGBM model, as a kind of grey prediction model, naturally has the advantages of grey system theory, but also there are some aspects to be improved. For example, the discussion of spatial stratified heterogeneity and temporal nonstationarity in grey forecasting models has not been presented. In the future, this issue will become a direction for further research and in-depth thinking. 

In addition, in the case analysis of this paper, although the LSTM model shows excellent fitting ability, for data series with small or insufficient sample size, under the condition of low training times, the LSTM model does not have the same advantage as the grey model in predicting the ability of small samples. Deep learning models such as LSTM are generally considered suitable for dealing with problems that have big data characteristics, while the grey model in this paper is aimed at uncertain systems with small samples. Although more population information can be obtained from China’s long-term population series, such behavior is not appropriate due to China’s population data being easily affected by national policies, such as the family planning policy, the two-child policy or the three-child policy. Therefore, the longer the population series of China is, the less reliable it is. Additionally, deep learning models that require big data seem to be unable to deal with the problem of population prediction in China well. However, as a current research hotspot, the deep learning model deserves us to seriously think about its integration and development with grey system theory in future research and it is a worthy direction to combine grey prediction models with some deep learning models to obtain more advanced results. In addition, in order to better solve the grey multivariate problem, it is also a valuable thought to extend the new cumulative operator to the high-dimensional grey model.

## Figures and Tables

**Table 1:** Summary of mainstream population prediction methods.

Method	Mechanism	Assumption	Merits	Drawbacks
Malthus model	Exponential model of population growth.	It is assumed that the change rate of the population is constant in an independent biological population.	The model has a deep historical background and it is suitable for short-term forecasting.	The model ignores the factors that have an important impact on the birth rate, such as limited living resources, space, productivity level and so on.
Leslie matrix model	This model is a difference equation in vector form and a population growth model grouped by age.	Assume the female fertility rate and mortality rate of all ages.	The method is classic and can clearly reflect the change in population development by age.	The model has high requirements for statistical data on age structure, and the changes in birth rate and mortality over time are not considered.
Logistic model	The population is predicted by solving the differential equation of population growth rate and population size.	Assume the population growth rate and maximum population capacity of the environment.	Considering the influence of environmental factors on population growth, the model has a simple structure and high prediction accuracy.	It struggles to deal with difficult nonlinear problems and the error fluctuation of long-term prediction is relatively large.
Markov chain model	A model of nondeterministic analysis in which the relationships between variables are given in the form of statistical values.	Under the condition that the present situation is known, the future situation of the system is only related to the present moment, not the past history.	The application range of the model is wide and the processing process is simple.	This model cannot make full use of existing information and is not suitable for long-term prediction.
Grey prediction model	The randomness of the original sequence is weakened by the accumulation operator and constructs the equation with partial difference and partial differential characteristics.	Population system is an uncertain system with only partial information known.	Small data requirements, simple modeling process and high prediction accuracy.	It is applicable to short-term and medium-term forecasts, and the data need to conform to the indexation trend.
Mathematical statistics method	Use mathematics and statistics knowledge to establish population prediction equation.	A priori condition of population information is required.	The theoretical basis of the model is completed and the prediction accuracy is high.	The modeling conditions are strict, the model is complex and it requires prior assumptions.
Time series prediction model	Uses the continuous law of the development of objective things, through statistical analysis to further predict the future development trend of the market.	There is no need to make model assumptions.	The time series prediction method is suitable for short- and medium-term prediction. It is very convenient to implement the model by computer.	When the historical data fluctuate greatly, the prediction results tend to have large errors.
BP neural network model	A computing system is established by simulating human brain neural tissue, and a large number of processing units are widely interconnected to form a network system.	There is no need to make model assumptions, just input historical data, since it has the basic characteristics of biological nervous system, it can learn data features and component models by algorithm.	The model has high self-learning and adaptive ability, strong generalization ability and high precision in the case of large amounts of data.	When the number of samples is not enough, the error of prediction is very large and the convergence speed of the algorithm is slow.

**Table 2:** Comparison between Logistic model and DA–NGBM model of registered population of Shanghai.

Year	Actual Data (Ten Thousand People)	Logistic Model		DA–NGBM Model	
		Fitting Data	MAPE (%)	Fitting Data	MAPE (%)
2011	1419.36	1425.82	0.45	1419.57	0.01
2012	1426.93	1432.83	0.42	1426.10	0.06
2013	1432.34	1439.25	0.48	1432.29	0.00
2014	1438.69	1445.11	0.45	1438.29	0.03
2015	1442.97	1450.46	0.52	1444.18	0.08
2016	1450.00	1455.35	0.37	1449.99	0.00
2017	1455.13	1459.80	0.33	1455.74	0.04
2018	1462.38	1463.87	0.13	1461.46	0.06
Simulation MAPE (%)		0.39		0.04	
2019	1469.30	1467.57	0.12	1468.84	0.15
2020	1475.63	1470.94	0.32	1475.29	0.19
Prediction MAPE (%)		0.22		0.17	

**Table 3:** Comparison of fitting and predicted birth rate in China from 2011 to 2020.

Year	Actual Data (%)	NGBM Model		DA–NGBM Model		GM (1,1) Model		DA–GM (1,1) Model		LSTM		Exponential Smoothing	
		Fitting Data (%)	MAPE (%)	Fitting Data (%)	MAPE (%)	Fitting Data (%)	MAPE (%)	Fitting Data (%)	MAPE (%)	Fitting Data (%)	MAPE (%)	Fitting Data (%)	MAPE (%)
2011	13.27	13.27	0.00	13.27	0.00	13.27	0.00	13.27	0.00	-	-	13.27	0.00
2012	14.57	14.57	0.00	14.57	0.00	14.25	2.17	14.57	0.00	-	-	13.27	8.92
2013	13.03	13.69	5.07	14.33	9.98	13.79	5.81	14.00	7.44	13.03	0.01	13.65	4.76
2014	13.83	13.14	4.99	13.83	0.00	13.34	3.58	13.46	2.68	13.83	0.03	13.47	2.61
2015	11.99	12.73	6.16	13.22	10.26	12.90	7.57	12.93	7.84	11.99	0.01	13.57	13.22
2016	13.57	12.40	8.65	12.58	7.30	12.48	8.07	12.43	8.40	13.58	0.07	13.11	3.39
2017	12.64	12.11	4.16	11.93	5.62	12.07	4.54	11.95	5.46	12.65	0.11	13.25	4.79
2018	10.86	11.87	9.27	11.28	3.87	11.67	7.46	11.48	5.71	10.87	0.14	13.07	20.33
Simulation MAPE (%)		5.47		4.63		5.60		4.70		**0.06**		7.25	
2019	10.41	11.65	11.87	10.66	**2.40**	11.29	8.45	11.04	6.05	10.82	3.93	12.42	19.31
2020	8.52	11.44	34.32	10.05	**17.96**	10.92	28.17	10.61	24.53	10.65	25.00	11.83	38.85

In the table, ‘-’ indicates that the value is missing in the calculation of LSTM model and the MAPE value in the table may have a slight error due to the reservation of decimals. The minimum error of the fitting results and the predicted results have been shown in bold and underlined.

**Table 4:** Comparison of fitting and predicted values of registered population in Jiangsu Province from 2011 to 2020.

Year	Actual Data (Ten Thousand People)	NGBM Model		DA–NGBM Model		GM(1,1) Model		DA–GM(1,1) Model		LSTM		Exponential Smoothing	
		Fitting Data	MAPE (%)	Fitting Data	MAPE (%)	Fitting Data	MAPE (%)	Fitting Data	MAPE (%)	Fitting Data	MAPE (%)	Fitting Data	MAPE (%)
2011	7514.25	7514.25	0.00	7514.25	0.00	7514.25	0.00	7514.25	0.00	-	-	7514.25	0.00
2012	7553.48	7553.48	0.00	7553.20	0.00	7574.25	0.28	7576.12	0.30	-	-	7514.25	0.52
2013	7616.84	7622.81	0.08	7620.62	0.05	7619.25	0.03	7619.24	0.03	7590.50	0.35	7553.48	0.83
2014	7684.69	7675.89	0.11	7672.74	0.16	7664.52	0.26	7662.61	0.29	7697.08	0.16	7616.84	0.88
2015	7717.59	7721.23	0.05	7717.60	0.00	7710.05	0.10	7706.22	0.15	7741.72	0.31	7684.69	0.43
2016	7775.66	7762.04	0.18	7758.19	0.22	7755.86	0.25	7750.08	0.33	7772.79	0.04	7717.59	0.75
2017	7794.19	7799.87	0.07	7796.00	0.02	7801.94	0.10	7794.19	0.00	7797.58	0.04	7775.66	0.24
2018	7831.86	7835.60	0.05	7831.86	0.00	7848.29	0.21	7838.56	0.09	7818.52	0.17	7794.19	0.48
Simulation MAPE (%)		0.08		**0.06**		0.18		0.15		0.18		0.52	
2019	7858.27	7869.78	0.14	7866.29	**0.10**	7894.91	0.47	7883.17	0.32	7836.66	0.27	7831.86	0.34
2020	7876.75	7902.77	0.33	7899.62	**0.29**	7941.82	0.83	7928.04	0.65	7852.67	0.31	7858.27	0.23

In the table, ‘-’ indicates that the value is missing in the calculation of LSTM model and the MAPE value in the table may have a slight error due to the reservation of decimals. The minimum error of the fitting results and the predicted results have been shown in bold and underlined.

**Table 5:** Comparison of fitting and predicted values of the male population of Nanjing city from 2011 to 2020.

Year	Actual Data (Ten Thousand People)	NGBM Model		DA–NGBM Model		GM(1,1) Model		DA–GM(1,1) Model		LSTM		Exponential Smoothing	
		Fitting Data	MAPE (%)	Fitting Data	MAPE (%)	Fitting Data	MAPE (%)	Fitting Data	MAPE (%)	Fitting Data	MAPE (%)	Fitting Data	MAPE (%)
2011	320.90	320.90	0.00	320.90	0.00	320.90	0.00	320.90	0.00	-	-	320.90	0.00
2012	321.39	321.39	0.00	321.39	0.00	318.16	1.00	318.16	1.00	-	-	320.90	0.15
2013	322.90	321.66	0.38	321.49	0.44	322.17	0.23	322.17	0.23	322.95	0.01	321.39	0.47
2014	325.04	324.52	0.16	324.33	0.22	326.22	0.36	326.22	0.36	326.13	0.33	322.90	0.66
2015	326.78	328.64	0.57	328.48	0.52	330.33	1.09	330.33	1.09	327.01	0.07	325.04	0.53
2016	330.86	333.53	0.81	333.47	0.79	334.49	1.10	334.49	1.10	330.38	0.15	326.78	1.23
2017	339.27	338.99	0.08	339.05	0.06	338.70	0.17	338.70	0.17	339.88	0.18	330.86	2.48
2018	346.85	344.90	0.56	345.10	0.51	342.97	1.12	342.97	1.12	345.40	0.42	339.27	2.19
Simulation MAPE (%)		0.37		0.32		0.63		0.63		**0.19**		0.96	
2019	352.80	351.17	0.46	351.53	**0.36**	347.28	1.56	347.28	1.56	346.65	1.74	346.85	1.69
2020	358.64	357.77	0.24	358.31	**0.09**	351.66	1.95	351.66	1.95	346.91	3.27	352.80	1.63

In the table, ‘-’ indicates that the value is missing in the calculation of LSTM model and the MAPE value in the table may have a slight error due to the reservation of decimals. The minimum error of the fitting results and the predicted results have been shown in bold and underlined.

## Data Availability

The datasets of this paper are available from the corresponding author upon reasonable request.
